# Motor Deficits and Cerebellar Atrophy in *Elovl5* Knock Out Mice

**DOI:** 10.3389/fncel.2017.00343

**Published:** 2017-10-30

**Authors:** Eriola Hoxha, Rebecca M. C. Gabriele, Ilaria Balbo, Francesco Ravera, Linda Masante, Vanessa Zambelli, Cristian Albergo, Nico Mitro, Donatella Caruso, Eleonora Di Gregorio, Alfredo Brusco, Barbara Borroni, Filippo Tempia

**Affiliations:** ^1^Neurophysiology of Neurodegenerative Diseases, Neuroscience Institute Cavalieri Ottolenghi (NICO), Torino, Italy; ^2^Department of Neuroscience, University of Torino, Torino, Italy; ^3^Department of Pharmacological and Biomolecular Sciences, Università degli Studi di Milano, Milan, Italy; ^4^Medical Genetics Unit, Città della Salute e della Scienza Hospital and Department of Medical Sciences, University of Torino, Torino, Italy; ^5^Neurology Unit, Department of Clinical and Experimental Sciences, University of Brescia, Brescia, Italy; ^6^National Institute of Neuroscience, Torino, Italy

**Keywords:** ELOVL5, spinocerebellar ataxia type 38 (SCA38), hyposmia, motor deficit, Purkinje cell, dendrites, dendritic spines, cerebellar atrophy

## Abstract

Spino-Cerebellar-Ataxia type 38 (SCA38) is caused by missense mutations in the very long chain fatty acid elongase 5 gene, *ELOVL5*. The main clinical findings in this disease are ataxia, hyposmia and cerebellar atrophy. Mice in which *Elovl5* has been knocked out represent a model of the loss of function hypothesis of SCA38. In agreement with this hypothesis, *Elovl5* knock out mice reproduced the main symptoms of patients, motor deficits at the beam balance test and hyposmia. The cerebellar cortex of *Elovl5* knock out mice showed a reduction of thickness of the molecular layer, already detectable at 6 months of age, confirmed at 12 and 18 months. The total perimeter length of the Purkinje cell (PC) layer was also reduced in *Elovl5* knock out mice. Since Elovl5 transcripts are expressed by PCs, whose dendrites are a major component of the molecular layer, we hypothesized that an alteration of their dendrites might be responsible for the reduced thickness of this layer. Reconstruction of the dendritic tree of biocytin-filled PCs, followed by Sholl analysis, showed that the distribution of distal dendrites was significantly reduced in Elovl5 knock out mice. Dendritic spine density was conserved. These results suggest that *Elovl5* knock out mice recapitulate SCA38 symptoms and that their cerebellar atrophy is due, at least in part, to a reduced extension of PC dendritic arborization.

## Introduction

The Spino-Cerebellar-Ataxia type 38 (SCA38) is caused by missense mutations in the very long chain fatty acid elongase 5 gene, *ELOVL5* (Di Gregorio et al., 2014). SCA38 patients show a progressive motor impairment starting with gait ataxia, nystagmus and a reduction of olfactory function (hyposmia; Borroni et al., 2016). At the magnetic resonance imaging (MRI), mild cerebellar atrophy is present, and functional positron electron tomography (FDG-PET) shows selective hypometabolism of the cerebellum, more prominent in the vermis (Borroni et al., 2016).

The ELOVL5 protein belongs to a family of enzymes that elongate very long-chain (>16 C) fatty acids, which are localized in the endoplasmic reticulum. Seven members of this family have been described in humans and mice (ELOVL1–7), each with substrate specificity towards acyl-CoA (Kihara, 2012). ELOVL1, 3, 6 and 7 are involved in elongation of saturated and mono-unsaturated fatty acids. ELOVL2, 4 and 5 are selective for polyunsaturated fatty acids (PUFA). Among the different *ELOVL* genes, *ELOVL1*, *5* and *6* are ubiquitously expressed whereas *ELOVL2, 3, 4* and *7* show a more specific tissue expression (Guillou et al., 2010). In addition to *ELOVL5*, also *ELOVL4* has been shown associated to a spinocerebellar ataxia (SCA34; Cadieux-Dion et al., 2014; Bourassa et al., 2015; Ozaki et al., 2015). The cellular mechanism of SCA34 and SCA38 is currently unknown.

**Figure 1:**
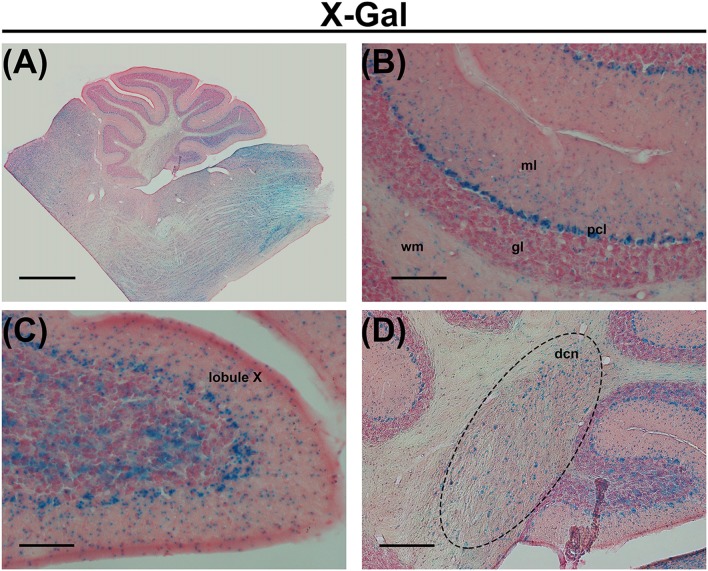
**(A)** X-Gal staining of a cerebellar slice of an *Elovl5^−/−^* mouse. **(B)** Higher magnification of the cerebellar cortex, showing X-Gal positive cells in the deeper part of the molecular layer, Purkinje cells (PCs), sparse cells in the granule cell layer and cells in the white matter. **(C)** Higher magnification of the lobule X showing X-Gal positive cells also in the granular layer. **(D)** Higher magnification of deep cerebellar nuclei showing X-Gal positive cells. ml (molecular layer); pcl (PC layer); gl (granular layer); wm (white matter); dcn (deep cerebellar nuclei). Calibration bars: **(A)** 1000 μm; **(B,C)** 100 μm; **(D)** 200 μm.

In the central nervous system, the most enriched PUFAs produced by the ELOVL5 pathway include arachidonic acid (AA, 20:4, ω6), eicosapentaenoic acid (EPA, 20:5, ω3) and docosahexaenoic acid (DHA, 22:6, ω3). These PUFAs are reduced in the serum of patients with SCA38, indicating a loss of function of the mutated *ELOVL5* allele (Di Gregorio et al., 2014), even if a toxic gain of function of the mutated protein cannot be excluded. In the cerebellum, *Elovl5* transcripts are expressed by cerebellar Purkinje cells (PCs), neurons of the deep cerebellar nuclei, cells in the white matter, small cells in the deeper part of the molecular layer and sparse cells in the granular layer (Di Gregorio et al., 2014; Figure 1). Several brainstem nuclei and mitral cells of the olfactory bulb also express high levels of Elovl5 (Figure 1 and Allen Mouse Brain Atlas). Such expression pattern is in line with the principal SCA38 symptoms, which are cerebellar ataxia and anosmia (Borroni et al., 2016). In cerebellar PCs, the ELOVL5 protein is localized in the soma and proximal dendrites (Di Gregorio et al., 2014), but the products of its activity can be delivered to other cellular regions including axon, dendrites and dendritic spines. These data suggest that a lack of ELOVL5 activity might damage cerebellar PCs, thereby causing ataxic symptoms of SCA38. However, it is not known which cellular structures of PCs are affected.

Mice with a deletion of the *Elovl5* gene (*Elovl5*^−/−^; Moon et al., 2009) might represent a model for the loss of function hypothesis, but their neural functions have not been tested yet. The aim of this study was to investigate, in such *Elovl5*^−/−^ mice, the consequences of *Elovl5* deletion by assessing motor performance and olfactory function and to search for cerebellar and PC structural defects.

## Materials and Methods

### Animals

All animal experimental procedures have been approved by the Ethical Committee of the University of Torino and authorized by the Italian Ministry of Health (authorization number: 161/2016-PR).

*Elovl5* knockout mice have been kindly provided by Dr. Moon and Dr. Horton of the UT Southwestern Medical Center (Moon et al., 2009) and bred in our Animal Facility at NICO.

*Elovl5*^−/−^ and wild type mice (the background of the mice is C57Bl6) were used for all the experimental paradigms, while heterozygous mice were only used as breeders. Data from female and male mice were pooled together because they showed no significant difference. Both *Elovl5*^−/−^ mice and their wild type littermates were kept on an natural diet without animal derivatives, containing essential PUFAs like linoleic and α-linolenic acids, but excluding the presence of omega-3 and omega-6 PUFAs downstream Elovl5, such as DHA, EPA and AA (Teklad Global 18% Protein Rodent Diet, Harlan Laboratories).

### X-Gal Staining

To perform the staining for β-galactosidase activity, we used 30 μm-thick sagittal slices prepared as for immunohistochemical reactions (see “Materials and Methods” section in the manuscript for more details). The floating slices were incubated overnight at 37°C with a solution containing 1 mg/ml X-gal, 0.02% Nonidet P-40, 0.01% sodium deoxycholate, 2 mM MgCl_2_, 5 mM potassium ferricyanide, and 5 mM potassium ferrocyanide (pH 7.5). Subsequently, the sections were mounted on gelatin-coated slides and let air dry overnight. The next day the sections were counter-stained with Nuclear Fast Red. The staining was performed as follows: mounted series were washed for 2 min in distilled water and then stained in 0.1% NFR (nuclear fast red) solution for 2 min. Following, sections were rinsed again in distilled water for 2 min, and then dehydrated using a series of alcohols: 50% (2 min), 75% (2 min), 90% (2 min) and 100% (2 min). Next, the gelatin-coated slides were immersed in xylene for 5 min and finally a clear glass coverslip was applied using a permanent mounting medium.

### Motor Tests

Motor tests were performed on 3, 6, 9 and 12 months old *Elovl5^−/−^* and wild type mice. All the experiments were conducted in the morning and under the same illumination conditions for all animals. The time schedule of motor tests was the following: for 3 days the mice were trained for the beam test. After 2 days of rest, the next day they started both rotarod, beam and footprinting tests. The beam test was continued for three consecutive days, together with the rotarod test, which was performed for other 2 days, so that the rotarod test lasted five consecutive days. All analyses have been done with the experimenter blind relative to the mouse genotype.

#### Balance Beam Test

To analyze balance and motor coordination, we performed the balance beam test on *Elovl5*^−/−^ and wild type mice. The beam test was performed as previously described (Hoxha et al., 2012). Briefly, we used a metal beam 1 cm wide and 100 cm long suspended 12 cm above the bench. The mice had to cross the beam to reach a cage enriched with toys. To allow the mice to familiarize with the experimental apparatus, thus reducing the stress due to an unknown procedure, the experiment was preceded by 3 days of habituation. On the day of the test, the animals were placed to acclimate in the behavioral room at least 15 min before the experiment. Both *Elovl5*^−/−^ and wild type mice were tested individually and each animal was encouraged to traverse the beam at least three times. The test was repeated for three consecutive days. The test was recorded using a video camera and analyzed offline by an operator blind to the genotype. The performance was assessed by measuring the latency required to cross the beam as well as the number of slips.

#### Accelerated Rotarod Test

We assessed the locomotor function using the accelerated rotarod test. Mice were tested for five consecutive days and then again 5 days later (10th day). This protocol was repeated at different ages, at 3, 6, 9 and 12 months, to evaluate long-term retention of the motor improvements. In each day, after 1 min training session at a constant speed (4 rpm), mice received three test sessions (with a minute interval between sessions) in which the rod (Mouse Rota-Rod, Ugo Basile Biological Research Apparatus, Comerio, Italy) accelerated continuously from 4 to 65 rpm with an acceleration of 5.5 rpm. The latency to fall off the rod was recorded. The cutoff in this experiment was set to 300 s.

#### Footprint Analysis

To analyze gait parameters, we performed the footprinting test. The test was performed as in Hoxha et al. (2013). We used a transparent Plexiglass walkway (20 cm high, 67 cm long and 4 cm wide) elevated 70 cm from the floor. A digital camera was placed underneath the clear platform and video recordings of the mice walking were collected. Elovl5^−/−^ and wild type mice were encouraged to walk at least for three consecutive steps per crossing. Still-frame from the recordings were extracted and analyzed offline using the Fiji software in order to obtain data concerning stride length (the distance of the same paw in a consecutive step), width (the distance between the center of the two hind or fore paws), the distance between ipsilateral fore paw and hind paw placements and the fore and hind stance. The data of the footprinting test are presented as box and whiskers plot (the box extends from the 25th to 75th percentiles and the whiskers represent the 10th and the 90th percentiles).

### Olfactory Test

We used an olfactory test to evaluate the ability of *Elovl5^−/−^* mice to smell volatile odors because one of the presenting clinical features of SCA38 patients is hyposmia (Borroni et al., 2016). We tested *Elovl5*^−/−^ and wild type mice at 3- (mice: wild type *n* = 16; *Elovl5*^−/−^ *n* = 11), 6- (mice: wild type *n* = 22; *Elovl5*^−/−^ *n* = 17), and 12-month of age (mice: wild type *n* = 24; *Elovl5*^−/−^ *n* = 19). The test measures the latency to localize a piece of appetizing food buried beneath a layer of cage bedding. We followed a previously described protocol (Yang and Crawley, 2009), with few minor adjustments. Before conducting the experiment it was necessary to identify a tempting food that could be used as an odorant stimulus.

We found that *Elovl5^−/−^* and wild type mice enjoyed Vitasnella ^®^ cookies with red berries. After separating mice in individual cages, odor familiarization was established by positioning a piece of cookie in each cage. The familiarization step lasted for 2 or 3 consecutive days. The cookies were placed in the afternoon on the cage bedding in the back-left corner and the following morning each cage was check to see whether they had been consumed. If not, the mouse was allowed to familiarize with the food for one more day. In case the mouse did not consume the cookie after a 3-day familiarization, it was excluded from the experiment. Following the familiarization step, the mice were subjected to a pre-test food deprivation, which lasted between 18 and 24 h prior to the test. This was important to motivate the animal to search for food. On the day of the experiment, mice were placed in the experimental room for an hour to acclimate to the novel environment. Following, the mouse was placed in the experimental cage, a clear clean cage without grid, containing about 3 cm of clean bedding. The mouse was left to explore the cage for 5 min. The mouse was then transferred into a temporary clean cage to allow the operator to bury the piece of cookie in the experimental cage. The cookie was placed about 1 cm below the bedding surface. The mouse was then placed back in the experimental cage, and the latency, considered as the time used by the mouse to dig out the cookie, was scored using a timer. The operator observed the experiment from behind a dark curtain inside the testing room and the experiment was recorded with a video camera. Mice were tested individually and the operator was blinded to the animal’s genotype. Following the test, the animals were placed back in their home cage with *ad libitum* access to their diet. The cookies during familiarization and testing were never positioned in the same corner to avoid false results.

### Morphological Characterization

#### Histological Procedures

We analyzed *Elovl5^−/−^* and wild type mice of 6, 12 and 18 months of age. Animals were anesthetized using a cocktail of ketamine (100 mg/kg body weight) and xylazine (10 mg/kg body weight) via intraperitoneal injection. The mice were intracardially perfused initially with a physiological solution (NaCl 0.9%) and then with 4% paraformaldehyde in 0.12 M phosphate buffer, pH 7.2–7.4. Following perfusion, the brains were removed and stored at 4°C for 24 h immersed in the same fixative. The brains were then transferred to a cryoprotectant solution made of 30% sucrose in 0.12 M phosphate buffer for few days. For each mouse, the cerebellum was separated and embedded in optimal cutting temperature compound, frozen in ice-cold isopentane. Samples were stored at −80°C until sectioning. Cerebella were serially cut by a cryostat in 30 μm-thick sagittal slices and collected in phosphate buffered saline (PBS).

Cresyl Violet Staining (Nissl Staining) was performed on one series for each *Elovl5^−/−^* and wild type mice at 6, 12 and 18 months of age. Free-floating sections were washed twice in PBS (15 min each). The series were mounted on gelatin coated slides and let air dry overnight. The staining was performed as follows: mounted series were washed for 2 min in distilled water to remove any residual salts and then stained in 0.1% Cresyl violet solution for 15 min. Following, sections were rinsed again in distilled water for 1 min, and then dehydrated using a series of alcohols: 50% (1 min), 70% (2 min), 95% (I) (2 min), 95% (II) (few seconds) and 100% (1 min). Next, the gelatin-coated slides were immersed in xylene for 5 min and finally a clear glass coverslip was applied using a permanent mounting medium. It should be noted that quantitative measurements might be not comparable to others reports, in which the tissues didn’t undergo shrinkage due to the dehydrating and drying steps.

#### Purkinje Cell Density and Molecular and Granular Layer Thickness

The density of PCs was evaluated in all cerebellar lobules of *Elovl5^−/−^* and wild type mice at different ages (6, 12, 18 months old) in anti-calbindin immunostained sections using Neurolucida software (MicroBrightField, Colchester, VT, USA) connected to an E-800 Nikon microscope under a 20× objective. At least three slices/animal and three animals/time point were analyzed. The density of PCs (expressed as number of PCs/mm) was obtained by drawing the outline of PC layer and marking the position of every labeled cell. For the PC layer length, we analyzed the total perimeter of the PC layer in the vermal slices.

The thickness (T) of the cerebellar molecular and granular layer was determined on the whole extent of each lobule, on Nissl stained slices by using Neurolucida software connected to an E-800 Nikon microscope with a 20× objective. To measure the thickness of the cerebellar layers we used the formula *T* = 2A/b_1_ + b_2_, where “A” was the area defined by the outer and inner profile of the molecular/granular layer, and “b_1_” and “b_2_” were the lengths of the two profiles. All measurements were done blind relative to the mouse genotype.

### Purkinje Cell Morphology

To verify alterations of dendritic arborization, we performed Sholl analysis on biocytin filled PCs localized at the apex of a cerebellar lobule. Briefly, the animals were anesthetized with isoflurane (Isoflurane-Vet, Merial, Italy) and decapitated. The cerebellar vermis was removed and transferred to an ice-cold artificial cerebrospinal fluid (ACSF) containing (in mM): 125 NaCl, 2.5 KCl, 2 CaCl_2_, 1 MgCl_2_, 1.25 NaH_2_PO_4_, 26 NaHCO_3_, 20 glucose, which was bubbled with 95% O_2_/5% CO_2_ (pH 7.4). Parasagittal cerebellar slices (200 μm thickness) were obtained using a vibratome (Leica Microsystems GmbH, Wetzlar, Germany) and kept for 1 h at 35°C. After recovery (1 h) at room temperature, single slices were placed in the recording chamber, which was perfused at a rate of 2–3 ml/min with ACSF bubbled with 95% O_2_/5% CO_2_. We filled individual PCs with a K-gluconate-based internal solution containing (in mM): 140 K-gluconate, 10 HEPES, 0.5 EGTA, 4 MgCl_2_, 4 Na_2_ATP, 0.4 Na_3_GTP and the pH was adjusted to 7.3 with KOH and filtered at 0.2 μm in which was added 0.5% biocytin. We kept the cells for 30 min in whole cell configuration to have a better distribution of biocytin in all dendritic branches of the PC. Then the slices were fixed in 4% paraformaldehyde in 0.12 M phosphate buffer, pH 7.2–7.4 for 15 min at room temperature. The slices were treated with a permeabilization buffer (0.1% Triton X-100, 0.5% Tween 20) for 20 min. The biocytin filled PC was visualized with Texas red-conjugated Avidin (1: 1000; Vector, Burlingame, CA, USA). The images were acquired using an E-800 Nikon microscope (Nikon, Melville, NY, USA) connected to a color CCD camera confocal microscope. The PC dendritic arbor was reconstructed manually and the Sholl analysis was done with Fiji software. We started our Sholl analysis 10 μm from the PC soma with a 22 μm radius. The number of intersections per radius was plotted. For the dendritic spine analysis we acquired confocal images with a Leica TCS SP5 confocal microscope with a 63× objective (Leica Microsystems, Wetzlar, Germany). The z-stack was acquired at 0.6 μm steps. The analysis was performed with Fiji software and the spines were counted in the optimal focal planes of the *z*-stack of confocal images. We performed spine analysis in the distal dendritic arbor of PCs. In each PC, three fields of the dendritic tree were analyzed. In each field, 15–20 spiny branchlets were measured. The number of spines/μm of dendritic length was provided as spine density. All measurements were done blind relative to the mouse genotype.

### Statistical Analysis

Comparisons were done with unpaired two tailed Student’s *t*-test or by two way or one way analyses of variance (ANOVA), followed by Holm-Sidak *post hoc* correction. Data of each animal were averaged and the statistics was performed between animals, except for Sholl analysis in which all reconstructed PCs for each genotype were compared.

## Results

### Impaired Motor Performance of *Elovl5^−/−^* Mice

To examine whether motor performance was altered in *Elovl5^−/−^* mice we performed different motor tests at different ages (3, 6 and 12 months). We found no sex-dependent differences in any behavioral test; therefore we grouped together male and female mice for the analysis.

**Figure 2:**
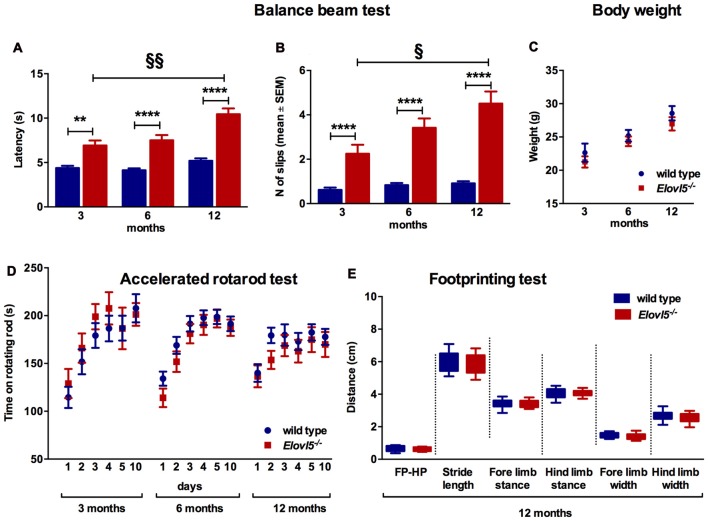
Impaired motor control in *Elovl5*^−/−^ mice** (A)** Increased latency to cross the beam for *Elovl5*^−/−^ compared to wild type mice (****p < 0.0001, **p < 0.01, *t*-test). **(B)** Increased mean number of foot-slips (****p < 0.0001). The performance of *Elovl5*^−/−^ mice worsened across months (^§§^*p* < 0.01, ^§^*p* < 0.05, one way analyses of variance (ANOVA)). **(C)** Comparable body weight between genotypes at all time points analyzed (*p* > 0.05, two way ANOVA). **(D)** The accelerated rotarod test repeated at 3, 6 and 12 months revealed a normal performance of *Elovl5*^−/−^ mice (*p* > 0.05, two way ANOVA). **(E)** The footprinting test performed at 12 months of age showed preserved gait parameters in *Elovl5*^−/−^ mice (*p* > 0.05, *t*-test). Values are mean ± SEM.

The balance beam test was performed to evaluate fine motor coordination and balance in *Elovl5^−/−^* and their wild type littermates. *Elovl5*^−/−^ mice showed a significantly longer latency to cross the beam compared to wild type mice (*p* < 0.0001; Two-way ANOVA: genotype effect, *F*_(1,145)_ = 103.1); for 3 months old mice: (*n* = 21 wild type: 4.38 ± 0.25; *n* = 13 *Elovl5*^−/−^: 6.92 ± 0.56), for 6 months old: (*n* = 39 wild type: 4.13 ± 0.21; *n* = 30 *Elovl5*^−/−^: 7.50 ± 0.59), and for 12 months old: (*n* = 30 wild type: 5.20 ± 0.27; *n* = 18 *Elovl5*^−/−^: 10.44 ± 0.65; Figure 2A). The average number of hindpaw slips from the beam was significantly greater for *Elovl5*^−/−^ mice compared to controls (*p* < 0.0001; Two-way ANOVA: genotype effect, *F*_(1,142)_ = 114.2); for 3 months old mice (*n* = 21 wild type: 0.62 ± 0.11; *n* = 13 *Elovl5*^−/−^: 2.25 ± 0.40), for 6 months old: (*n* = 38 wild type: 0.83 ± 0.1; *n* = 27 *Elovl5*^−/−^: 3.42 ± 0.42) and for 12 months old: (*n* = 30 wild type: 0.91 ± 0.09; *n* = 19 *Elovl5*^−/−^: 4.51 ± 0.55; Figure 2B). This motor deficit was progressive, since *Elovl5*^−/−^ mice worsened their performance with age increasing the latency to cross the beam from 3 to 12 months (*p* < 0.001; One-way ANOVA: *F*_(2,58)_ = 3.35) and doubling the number of slips from 3 to 12 months (*p* < 0.05, One way ANOVA: *F*_(2,56)_ = 2.96; Figure 2B). There was no significant difference in body weight across genotypes, so that effects of weight in the performance could be excluded (*p* > 0.05, Two-way ANOVA *F*_(1,119)_ = 2.223; Figure 2C). We performed the test also at 18 months of age. At this age *Elovl5*^−/−^ mice were unable to walk on a 1 cm wide beam while their wild type littermates could perform the task (data not shown). With a 2 cm beam also 18 months old *Elovl5*^−/−^ mice could cross from one side to the other, although with several footslips (data not shown).

We evaluated the locomotor skill and the retention of improvements in *Elovl5^−/−^* and wild type mice by means of the accelerated rotarod test. *Elovl5*^−/−^ mice did not show any sign of motor deficit at any age analyzed (*p* > 0.05; Two-way ANOVA: genotype effect, *F*_(1,1056)_ = 0.8046); for 3 month old mice: (*n* = 20 wild type and *n* = 11 *Elovl5*^−/−^), for 6 month old: (*n* = 55 wild type and *n* = 37 *Elovl5*^−/−^) and for 12 month old: (*n* = 33 wild type and *n* = 26 *Elovl5*^−/−^; Figure 2D).

The gait characteristics were evaluated by means of footprinting analysis using 12 month old mice of both genotypes (*n* = 22 wild type and *n* = 16 *Elovl5*^−/−^). Our analysis indicated that there was not a significant difference in the gait parameters across genotypes (for each gait parameter: *p* > 0.05, Unpaired Student’s *t*-test; Figure 2E; for FP-HP placement: wild type: 0.63 ± 0.04 and *Elovl5*^−/−^: 0.63 ± 0.03; for stride length: wild type: 5.99 ± 0.16 and *Elovl5*^−/−^: 5.94 ± 0.16; for fore limb stance: wild type: 3.37 ± 0.07 and *Elovl5*^−/−^: 3.38 ± 0.07; for hind limb stance: wild type: 4.05 ± 0.08 and *Elovl5*^−/−^: 4.07 ± 0.06; for fore limb width: wild type: 1.50 ± 0.04 and *Elovl5*^−/−^: 1.42 ± 0.06; for hind limb width: wild type: 2.67 ± 0.07 and *Elovl5*^−/−^: 2.57 ± 0.09). The coefficient of variation of all the parameters was comparable between genotypes (*p* > 0.05, Student’s *t*-test). The footprinting analysis was repeated in 18 month old mice, in which no significant difference was detected for any parameter (data not shown).

### *Elovl5*^−/−^ Mice Present Hyposmia

**Figure 3:**
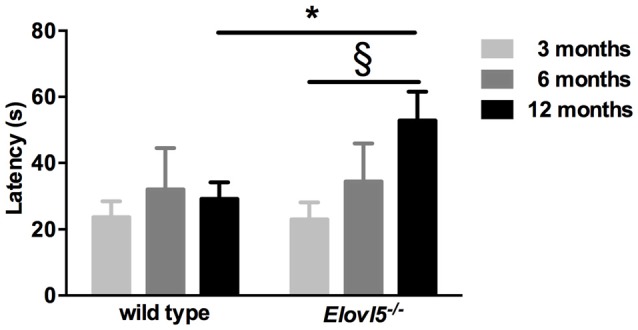
*Elovl5*^−/−^ mice show hyposmia. The latency to find the buried cookie is significantly longer for *Elovl5*^−/−^ mice compared to wild type animals at 12 months (black bars; *p < 0.05, *t*-test). At younger ages, no significant difference between genotypes was present (*p* > 0.05, *t*-test). *Comparison between genotypes; (§) comparison between 3 and 12 months of the same genotype. Values are mean ± SEM.

One of the presenting features in SCA38 patients is hyposmia (Borroni et al., 2016). To explore whether *Elovl5^−/−^* mice had a diminished olfaction we performed the buried food test. We tested both *Elovl5*^−/−^ and wild type animals at 3, 6 and 12 months. *Elovl5*^−/−^ displayed an increased latency to retrieve the cookie relative to wild type mice at 12 months of age (*p* < 0.05, Unpaired Student’s *t*-test; Figure 3). One way ANOVA analysis revealed that the olfaction diminished with the age in *Elovl5*^−/−^ mice (*p* < 0.05) while it remained preserved in wild type mice (*p* > 0.05, one way ANOVA).

### *Elovl5*^−/−^ Mice Display Cerebellar Atrophy

**Figure 4:**
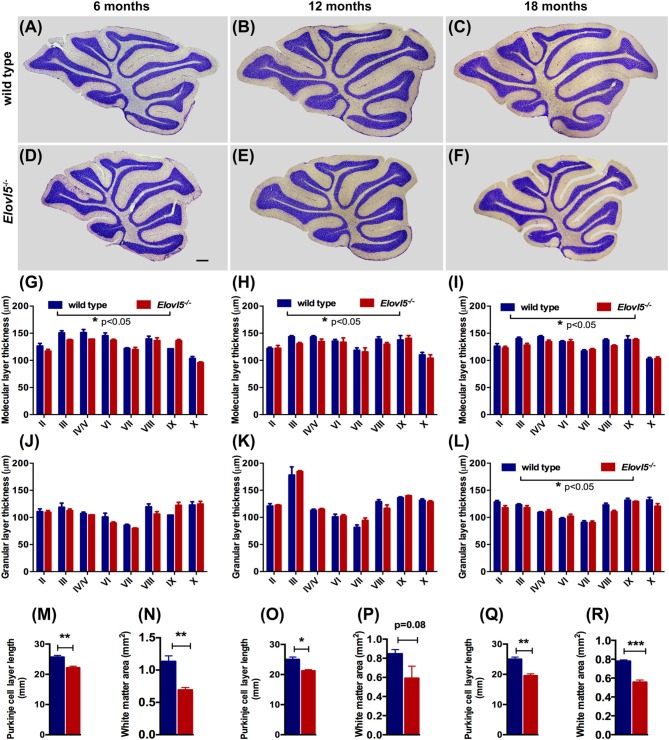
*Elovl5*^−/−^ mice show cerebellar atrophy. **(A–F)** Nissl-stained sagittal sections of the cerebellum from wild type **(A–C)** and *Elovl5*^−/−^ mice **(D–F)** at 6, 12 and 18 months of age. **(G–I)** Average thickness of the molecular layer and **(J–L)** granular layer of each cerebellar lobule for *Elovl5*^−/−^ (red) and wild type mice (blue) at 6, 12 and 18 months, respectively. **(M,O,Q)** Reduced total perimeter length of the PC layer and **(N,P,R)** white matter area in the cerebellum of *Elovl5*^−/−^ mice at all ages analyzed. *p < 0.05; **p < 0.01, ***p < 0.001 two way ANOVA. Results are reported as mean ± SEM. Scale bar 100 μm.

**Figure 5:**
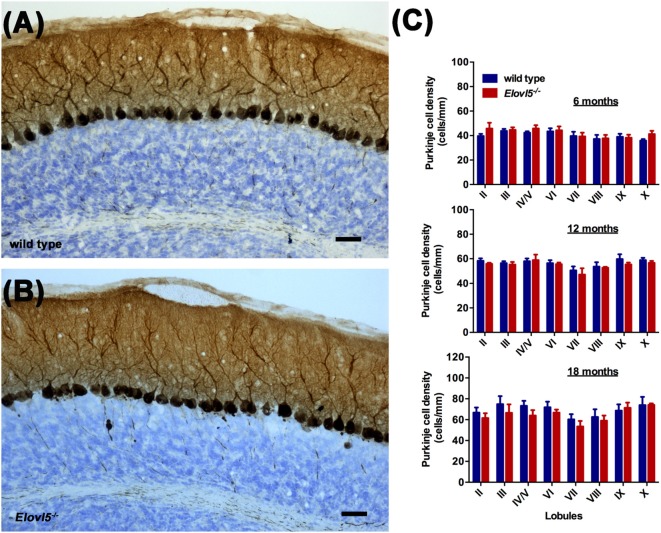
Normal PC density in the cerebellum of *Elovl5*^−/−^ mice. Calbindin staining of sagittal cerebellar sections obtained from **(A)** wild type and **(B)** *Elovl5*^−/−^ mice. **(C)** PC density analyzed at 6, 12 and 18 months of age in both genotypes. Scale bar 40 μm.

To investigate whether the motor impairment was associated with cerebellar degeneration, we measured PC density as well as the thickness of both granular and molecular layers at different time points (6, 12 and 18 months of age) in* Elovl5*^−/−^ mice and their wild type littermates. The cerebellum of *Elovl5*^−/−^ mice had a normal foliation and cytoarchitecture at all time points analyzed (Figures 4A–F). However, starting from 6 months of age there was a significant reduction in the molecular layer of the cerebellum of *Elovl5*^−/−^ mice (*p* < 0.05; Two-way ANOVA: genotype effect, *F*_(1,29)_ = 6.587; Figure 4G). *Post hoc* multiple comparison revealed a significant reduction in the 3rd and 4th/5th lobules (*p* < 0.05). On the other hand, no abnormalities of the granular layer were present at this age (*p* > 0.05; Two-way ANOVA *F*_(1,29)_ = 1.052; Figure 4J). At 12 months of age Two-way ANOVA revealed a significant overall change in the molecular layer thickness of* Elovl5*^−/−^ mice (*p* < 0.05, *F*_(1,60)_ = 4.027; Figure 4H). *Post hoc* Holm-Sidak multiple comparisons revealed a significant reduction of the molecular layer in the 3rd (*p* = 0.008; *t* = 2.798) and 8th lobules (*p* = 0.02, *t* = 2.411; Figure 4H). The granular layer was comparable between genotypes also at this age (*p* > 0.05; Two-way ANOVA *F*_(1,44)_ = 0.341; Figure 4K). Interestingly, at 18 months the molecular layer thickness decreased significantly in *Elovl5*^−/−^ mice compared to their wild type littermates (*p* < 0.05; Two-way ANOVA: genotype effect, *F*_(1,61)_ = 6.687; Figure 4I). *Post hoc* multiple comparison showed a specific reduction of the molecular layer of the 3rd lobule (*p* = 0.006; *t* = 2.835), for the 4th/5th lobule (*p* = 0.033; *t* = 2.185) and for the 8th lobule (*p* = 0.014; *t* = 2.519; Figure 4I). Despite the other ages analyzed, at 18 months of age there was a significant reduction also in the granular layer of *Elovl5*^−/−^ mice (*p* < 0.01; Two-way ANOVA: genotype effect, *F*_(1,61)_ = 7.594; Figure 4L). Unexpectedly, the PC density did not change at any time point analyzed (Figures 5A–C). However, we found a significant reduction of the total perimeter length of the PC layer at 6, 12 and 18 months in *Elovl5*^−/−^ compared to wild type mice (*p* < 0.05, Unpaired Student’s *t-test;* Figures 4M,O,Q). This decrease was paralleled to a significant reduction of the cerebellar white matter in *Elovl5*^−/−^ mice (*p* < 0.05, Unpaired Student’s *t-test;* Figures 4N,P,R). These cerebellar abnormalities are in accord with the impairment in motor performance observed in *Elovl5*^−/−^ mice.

### Reduced Purkinje Cell Dendritic Tree in *Elovl5^−/−^* Mice

**Figure 6:**
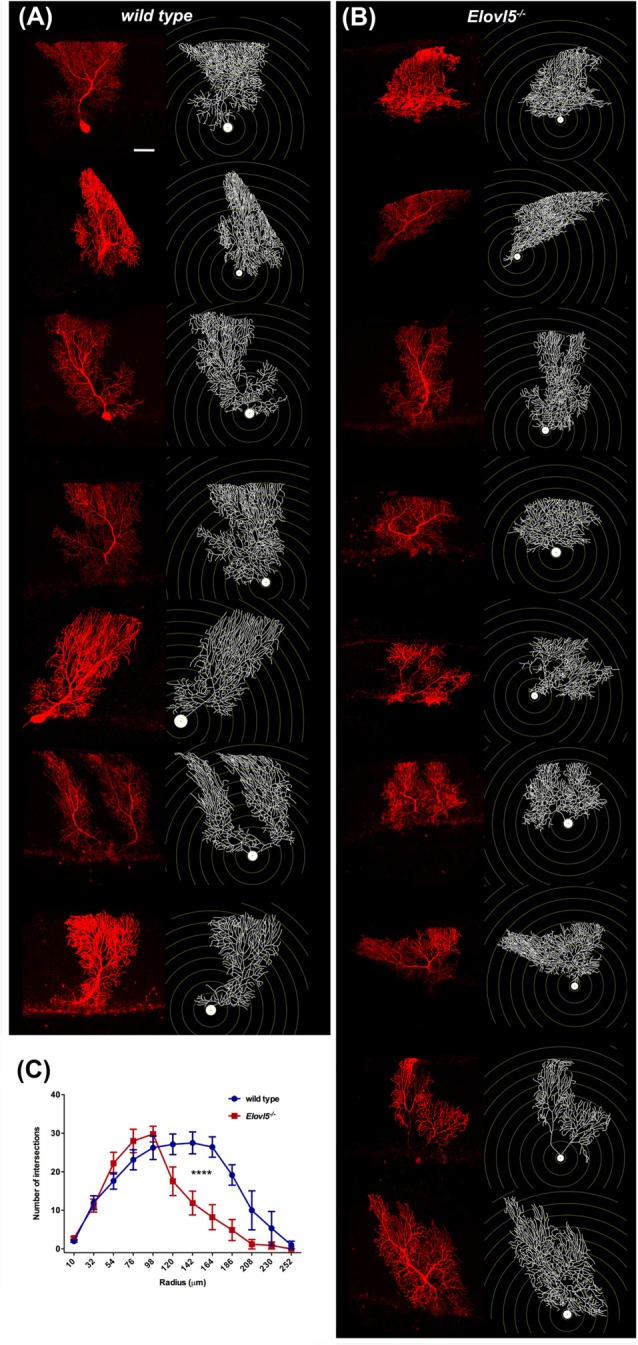
Reduced PC dendritic tree extension in *Elovl5*^−/−^ mice. Biocytin filled PCs and reconstructed dendritic structures for wild type **(A)** and *Elovl5*^−/−^ mice **(B)**. **(C)** Sholl plot graph showing a smaller number of intersections in the distal part of dendritic tree for *Elovl5*^−/−^ PCs, indicating a reduced extension the dendrites (cells: *n* = 9) relative to their wild type littermates (cells: *n* = 8; ****p < 0.001, two-way ANOVA). Scale bar 40 μm.

Cerebellar PCs, whose dendrites are a major component of the molecular layer, show a high expression of *Elovl5* (Figure 1). Therefore, we hypothesized that the reduction of the molecular layer could be attributed to an alteration of the PC dendritic tree. To this aim, we analyzed the morphology of PCs dendrites at 12 months of age. We filled the cells with biocytin and reconstructed their dendritic arbor (Figures 6A,B). The complexity of the dendritic arbor for *Elovl5*^−/−^ and wild type mice was assessed by Sholl analysis. Two way ANOVA analysis of the distribution of intersections as a function of the radius showed a significant difference between genotypes (*p* < 0.0001; Figure 6C). *Elovl5*^−/−^ PCs displayed normal distribution of the proximal dendrites while showed an alteration of the distribution of distal dendrites. *Elovl5*^−/−^ PCs showed a reduced number of intersections starting from 120 μm away from the soma, thus shifting the distribution to the left (Figure 6C). This result suggests that the reduced PC dendritic arbor might be the cause of the observed decrease of molecular layer thickness.

### Normal Purkinje Cell Dendritic Spine Density in *Elovl5^−/−^* Mice

**Figure 7:**
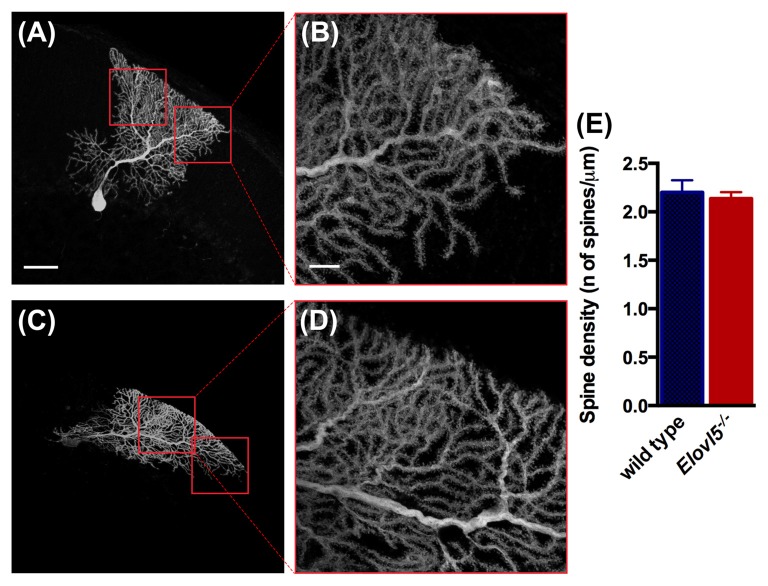
Normal number of spines in PC dendrites of *Elovl5*^−/−^ mice. **(A,C)** Representative PCs filled with biocytin from wild type and *Elovl5*^−/−^ mice, respectively. **(B,D)** Higher magnification images of the dendritic arbor from the cells represented in **(A,C)**. Scale bars: for **(A,C)** 40 μm; for **(B,D)** 10 μm. **(E)** Spine density obtained from the analysis of more than 15 dendrites per PC (number of cells: *n* = 7 wild type and *n* = 8 *Elovl5*^−/−^ PCs). Three animals per genotype were analyzed.

Given the reduced extension of the dendritic arbor of *Elovl5^−/−^* PCs, we investigated whether the number of dendritic spines was altered. We conducted our analysis in the distal part of the dendritic arbor of PCs (Figures 7A–D). We found a comparable number of dendritic spines between *Elovl5*^−/−^ and wild type PCs (cells, *n* = 7 wild type: 2.20 ± 0.13; *n* = 8 *Elovl5*^−/−^: 2.14 ± 0.07, *p* > 0.05 Unpaired Student’s *t*-test; Figure 7E). These results indicate that the reduced dendritic arbor extension is not accompanied by a change in the number of spines in *Elovl5*^−/−^ PCs.

## Discussion

Mice with a complete deletion of *Elovl5* (*Elovl5*^−/−^) display motor deficits and hyposmia. Indeed, the main symptoms of SCA38 patients with a mutation of *ELOVL5* are ataxia and olfactory deficit (Di Gregorio et al., 2014; Borroni et al., 2016). The results obtained in *Elovl5*^−/−^ mice are in line with the loss-of-function hypothesis of SCA38. However, in SCA38 patients the pathogenic mechanism might be more complex. *Elovl5*^−/−^ mice completely lack the Elovl5 protein, while patients with SCA38 have only one mutated allele, suggesting that also a gain of function of the mutated ELOVL5 protein might play a role.

*Elovl5*^−/−^ mice displayed a marked deficit in the beam balance test. On the contrary, the rotarod test did not reveal any significant difference between wild type and *Elovl5*^−/−^ mice. Since impairment on the beam test might be due to a vestibular deficit, we performed another test depending on vestibular function, the negative geotaxis test, but *Elovl5*^−/−^ mice didn’t show any impairment at this test (Supplementary Figures S1, S2). We argue that the motor tests might have a different sensitivity in revealing motor impairment, depending on the severity of ataxia and of the peculiar features of each ataxic syndrome. A phenotype similar to that of *Elovl5*^−/−^ mice was described by Larivière et al. (2015) in a murine model of autosomal recessive spastic ataxia of Charlevoix-Saguenay (*Sacs*^−/−^). The authors were able to detect the first signs of ataxia with the balance beam test but not with the rotarod test. On the other hand, Switonski et al. (2015) found an impaired performance of SCA3 mice in the rotarod test despite the absence of deficits in the balance beam test. The motor impairment observed in *Elovl5*^−/−^ mice is reminiscent of the initial stages of ataxia, especially in slowly progressing forms observed in spino-cerebellar ataxia (SCA) patients, in which instability can be revealed by tandem gait while regular walking is not affected (Bodranghien et al., 2016).

The cerebellum of patients with SCA38 in mild disease stages had a normal appearance at MRI, while in moderate to severe stages cerebellar atrophy became clearly visible (Di Gregorio et al., 2014; Borroni et al., 2016). In *Elovl5*^−/−^ mice, although the cerebellum was regularly formed and all lobules were preserved, a quantitative analysis revealed a cerebellar atrophy. In fact, we found that the total perimeter length of the PC layer was markedly reduced at all ages analyzed. Furthermore, this was accompanied by a reduction of the cerebellar white matter area. In addition, we found a thinning of the molecular layer. It is interesting to note that the extent of the molecular layer thinning was different between lobules. Indeed, a similar heterogeneity between lobules has been described in several other animal models (Tolbert et al., 1995; Lee et al., 2006; Perkins et al., 2016). It is well reported that the cerebellum has regional differences, including different packing density of the cells and different expression of specific markers within the lobules (for review, see Cerminara et al., 2015). The reason for the lobular variability in *Elovl5*^−/−^ mice is not known, but could be related to a different susceptibility of certain regions to Elovl5-dependent mechanisms. The molecular layer contains the dendritic trees of PCs, parallel fibers and inhibitory interneurons. Since *Elovl5* in the cerebellum is expressed by PCs, we hypothesized that the dendrites of these cells might be altered, thereby causing a reduction in the thickness of the molecular layer. In fact, the analysis of PC dendrites revealed a reduction in extension, which could be responsible for the thinning of the molecular layer.

Dendritic alterations are often accompanied by changes in dendritic spines, but in *Elovl5*^−/−^ mice the density of these structures in the distal part of the dendrites was normal. A reduced dendritic extension with conserved spine density has been observed also in other murine models of ataxia (Kodama et al., 2006; Benedetti et al., 2016). This result indicates that Elovl5 is not necessary for spine formation but is very important to maintain a correct distribution of the PC dendritic branches in the molecular layer.

At present, the role of Elovl5 or of PUFAs in the dendrites is not known. It is possible that long-chain PUFAs are required for normal plasma membrane fluidity or for the elongation of dendrites and that these functions become critical with aging. It is interesting to note that very similar defects of PC dendrites, namely a reduced extension with preserved spine density, have been reported in “rocker”mice with mutation of Cav2.1 voltage-gated calcium channels (Kodama et al., 2006) and in “lethargic” mice (Benedetti et al., 2016), which have a deletion of the gene encoding the β_4_ subunit of voltage-gated calcium channels. Future studies are necessary to know whether the dendritic defects in these two different models have any mechanism in common.

## Author Contributions

EH and FT performed and supervised the experiments and data analysis and wrote the manuscript. RMCG, IB, FR, LM, VZ and CA performed the experiments and data analysis. EH, FT, NM, DC, EDG, AB and BB contributed to the conception of the work and to the discussion of the results. All authors approved the final version of the manuscript.

## Conflict of Interest Statement

The authors declare that the research was conducted in the absence of any commercial or financial relationships that could be construed as a potential conflict of interest.
