# Supplementary material for: Motor Deficits and Cerebellar Atrophy in Elovl5 Knock Out Mice
Source: Front Cell Neurosci. 2017 Oct 30;11:343. doi: 10.3389/fncel.2017.00343 (PMC5670146; doi:10.3389/fncel.2017.00343)
Supplement: Supplementary file 1 [file Data_Sheet_1.doc]

**Supplementary Figure S1.** Geotaxis of 12 months old *Elovl5^-/-^* mice (n = 6) and their wild type littermates (n = 13). *Elovl5^-/-^* mice showed no deficits in geotaxis test (p > 0.05, t-test). Error bars indicate SEM.

**Supplementary Figure S2.** Images representing the absence of hindlimb clasping in 12 months old *Elovl5^-/-^* mice (B) and in their wild type littermates (A).

**Supplemental Experimental Procedures**

**Negative geotaxis.**

To test vestibular function we performed the negative geotaxis in 12 months old mice for both genotypes. The mice were placed in the middle of a platform, 30° inclined to the surface plane. The mice were positioned in a head down position and the latency to turn 180° to a head up position was measured.

**Hindlimb clasping test.**

We tested 12 months old *Elovl5^-/-^* mice (n = 6) and their wild type littermates (n = 13) in the hindlimb clasping test to further evaluate the phenotype (Guyenet et al., 2010). We grasped the tail near the base and lifted the mouse for 10 seconds. We observed the hindlimbs and assigned a score for each position: score 0: hindlimbs are splayed outward, away from the abdomen; score 1: one hindlimb is retracted toward the abdomen for more than 50% of the time of the observation; score 2: both hindlimbs are partially retracted toward the abdomen for more than 50% of the time; score 3: both hindlimbs are totally retracted toward the abdomen for more than 50% of the time

**Supplemental references.**

Guyenet SJ, Furrer SA, Damian VM, Baughan TD, La Spada AR, Garden GA. A simple composite phenotype scoring system for evaluating mouse models of cerebellar ataxia. J Vis Exp (2010) **21** pii: 1787. doi: 10.3791/1787.
