# Supplementary figures and images for: Motor Deficits and Cerebellar Atrophy in Elovl5 Knock Out Mice
Source: Front Cell Neurosci. 2017 Oct 30;11:343. doi: 10.3389/fncel.2017.00343 (PMC5670146; doi:10.3389/fncel.2017.00343)

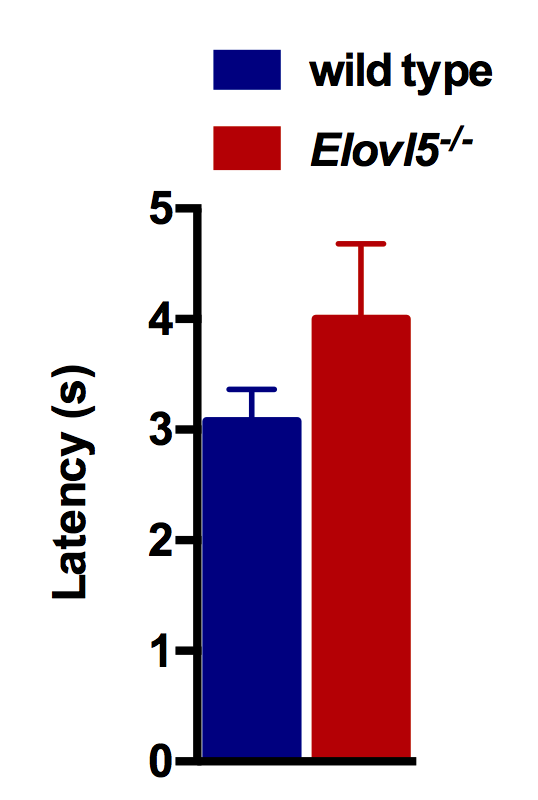

Supplement: Supplementary file 2 [file Image_1.tiff]

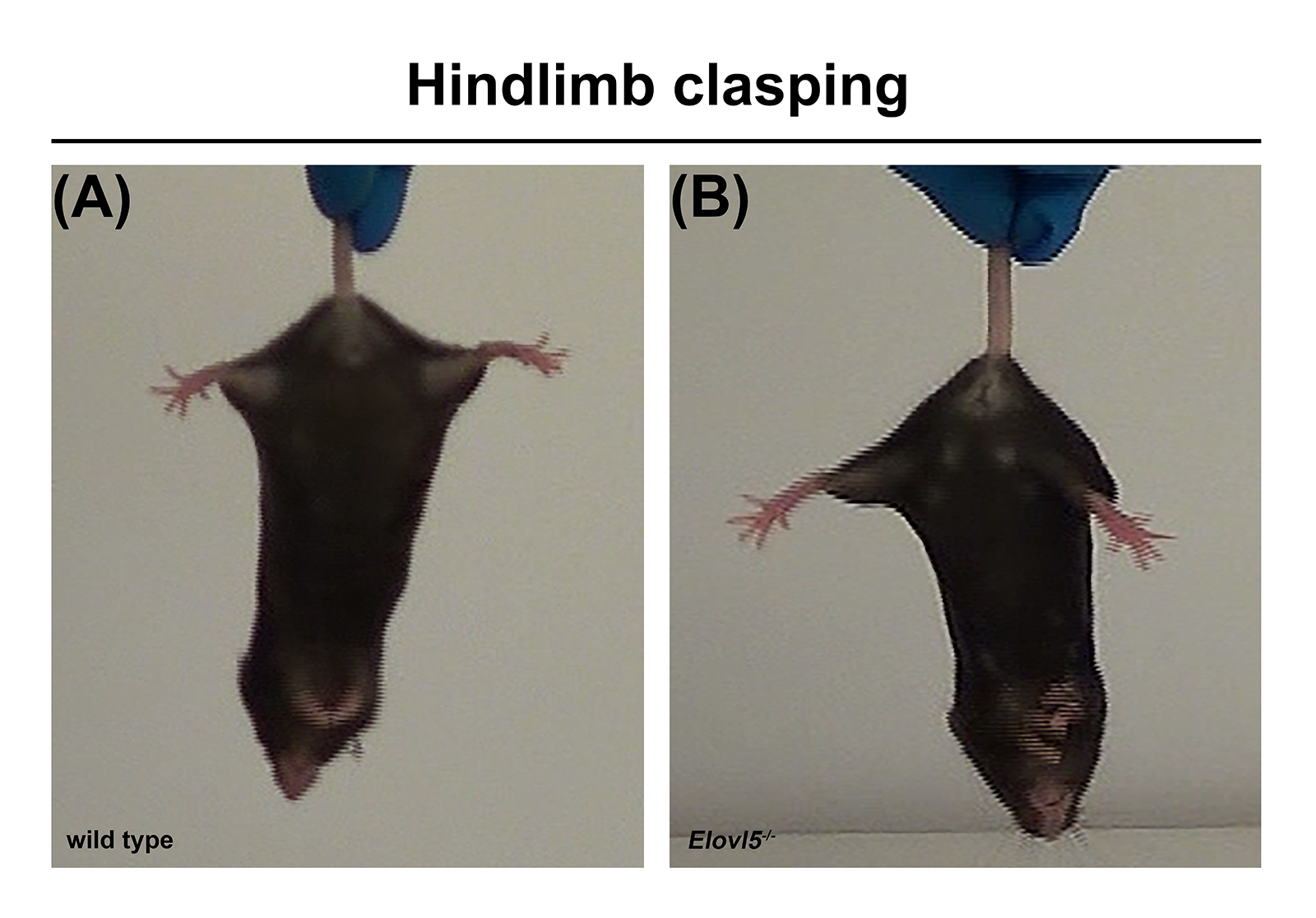

Supplement: Supplementary file 3 [file Image_2.tif]
